# COVID-19: a catalyst for the digitization of surgical teaching at a German University Hospital

**DOI:** 10.1186/s12909-022-03362-2

**Published:** 2022-04-22

**Authors:** Milan Anton Wolf, Antonius Pizanis, Gerrit Fischer, Frank Langer, Philipp Scherber, Janine Stutz, Marcel Orth, Tim Pohlemann, Tobias Fritz

**Affiliations:** 1 Department of Trauma, Hand and Reconstructive Surgery, Saarland University Medical Center, Kirrbergerstr. 100, 66421 Homburg, Germany; 2 Department of Orthopaedics and Orthopaedic Surgery, Saarland University Medical Center, Kirrbergerstr. 100, 66421 Homburg, Germany; 3 Department of Neurosurgery, Saarland University Medical Center, Kirrbergerstr. 100, 66421 Homburg, Germany; 4 Department of Thoracic and Cardiac Surgery, Saarland University Medical Center, Kirrbergerstr. 100, 66421 Homburg, Germany; 5 Department of General Surgery, Visceral, Vascular and Pediatric Surgery, Saarland University Medical Center, Kirrbergerstr. 100, 66421 Homburg, Germany

**Keywords:** Surgical education, eLearning, Covid-19, Webinar, Digitalization

## Abstract

**Background:**

The summer semester 2020, had to be restructured due to the SARS-CoV-2 pandemic and the associated contact restrictions. Here, for the first time, the established lectures in lecture halls and small group seminars could not be conducted in presence as usual. A possible tool for the implementation of medical teaching, offers the use of eLearning, online webinars and learning platforms. At present it is unclear how the SARS-CoV-2 pandemic will affect surgical teaching, how digitization will be accepted by students, and how virtual teaching can be expanded in the future.

**Methods:**

The teaching, which was previously delivered purely through face-to-face lectures, was completely converted to digital media. For this purpose, all lectures were recorded and were available to students on demand. The seminars were held as a twice a week occurring online webinar. The block internship was also conducted as a daily online webinar and concluded with an online exam at the end.

At the end of the semester, a survey of the students was carried out, which was answered by *n* = 192 students with an anonymized questionnaire. The questionnaire inquires about the previous and current experience with eLearning, as well as the possibility of a further development towards a purely digital university.

**Results:**

There were *n* = 192 students in the study population. For 88%, the conversion of classes to web-based lectures represented their first eLearning experience. For 77% of all students, the digitization of teaching led to a change in the way they prepare for class. 73% of the participating students are of the opinion that eLearning lectures should continue to be offered. 54% of the students felt that eLearning lectures made more sense than face-to-face lectures. A purely virtual university could be imagined by 41% of the students.

**Conclusion:**

The conversion of teaching represented the first contact with eLearning for most students. Overall, the eLearning offering was experienced as positive. Due to the new teaching structure, the way of learning had already changed during the semester. Based on the new eLearning content, the already existing formats can be further expanded in the future. Nevertheless, it turned out that the practical-surgical contents and skills cannot be adequately represented by purely online offers; for this, the development of hybrid practice-oriented teaching concepts is necessary.

**Supplementary Information:**

The online version contains supplementary material available at 10.1186/s12909-022-03362-2.

## Introduction

The year 2020 was substantially shaped by the effects of the viral pandemic and presented the world population with serious challenges in a variety of different ways. The effect of infection with the Sars-Cov-2 virus can lead to the disease Covid-19, which can have a severe and often fatal course, especially in pre-diseased and elderly patients [1–4]. Even with vaccines now available, as the risk from mutation of the pathogens increases, the most effective therapeutic measure to contain the virus is to reduce contact and adhere physical distance [5–7].

Especially with the current high demand for trained medical professionals, an adapted continuation of education is crucial. In most universities, students used to physically gather in small groups to discuss disease-specific problems and in lecture halls to gain practical and theoretical knowledge. Activities that are currently inconceivable and should be avoided at all costs for the protection of both trainees and society.

For years, there have been many ways of bypassing personal contact and imparting knowledge across wider distance, but in recent years these have only been used to a limited extent and mostly only additively at the majority of medical faculties. The pandemic has accelerated the use of these options [8]. Digital teaching offers are very suitable for continuing lectures virtually, as virtual lectures still allow a certain degree of interaction between the teacher and the students [9–11].

In addition to classical lectures, practical seminars in which practical knowledge such as physical examination techniques, anamnesis interviews and other hard skills are taught are an integral part of medical training. These courses were also not feasible in the usual way in 2020 and had to be replaced by a digital equivalent, which presented a particular hurdle for primarily practical subjects such as surgery.

To date, not all areas of application have been researched to determine which of the existing digital teaching concepts can be implemented as quickly, cost-effectively and comprehensively as possible and still provide a high didactic value. This study seeks to address the following questions: 1) can surgical teaching be converted to a digital teaching experience through the available teaching formats within the pandemic? 2) Is the transition being embraced by students? 3) Which methods should be adapted or supplemented despite the pandemic?

## Material and methods

### Study setting

In the context of this study, the main surgical lecture and the associated practical seminar as well as the block internship as an in-depth clinical part of surgical teaching—subdivided into the specialties of trauma surgery, general surgery, cardiothoracic surgery, neurosurgery and oral and maxillofacial surgery—were transferred from traditional teaching to a digital form at Saarland University Medical Center. The courses took place in the 3rd and 4th teaching years (7th and 8th semesters) and are mandatory for graduation and therefore cannot be suspended. Therefore, all face-to-face courses have been converted into a digital course.

The main lecture which is supposed to teach pathophysiological basics takes place over one semester with 5 teaching hours per week. The practical seminar, in which the first practical skills are learned, and specific clinical syndromes are dealt with in depth, takes place in the same semester with 4 teaching hours per week. The block internship, in which clinical examinations and advanced surgical skills are to be learned, takes place in the 8th semester over 2 weeks/ 80 h. Prior to the COVID-19 pandemic, all courses were face-to-face with no e-learning.

### Study design

At the end of the semester, this longitudinal study involved data collection from all participating students in the form of a digital questionnaire. Qualitative and quantitative questions were asked and analyzed as part of the questionnaire.

Participation in the study/questionnaire was anonymous and voluntary. Prior to the study, all participating students provided informed consent to the study. The study was performed in agreement with the responsible ethics committee (ID number of the Ethics Committee of the Saarland Medical Association: 221/21.").

### Data collection

At the end of the summer semester 2020, a digital questionnaire was sent to all students. Skipping and thus not answering individual questions was allowed. The response options of the questionnaire items represent 6 Likert-type scales. Some aspects and the baseline characteristics were addressed by polar questions. The questionnaire asked 20 questions about different aspects of digital teaching:

1. Baseline characteristics (3 questions).
2. Surgical skills and surgical career (2 questions)
3. Structure and requirement of eLearning (5 questions)
4. eLearning as a new medium (3 questions)
5. Media used (3 questions)
6. eLearning in the future (3 questions)
7. Quality of teaching (1 question)

At the end of the study, an abductive analysis of the results was carried out.

### Digitalization of surgical teaching

The main lecture was traditionally held as a class in a large lecture hall for all students of the semester at the same time. The lecture was led by an experienced lecturer (department chair or appropriate representative). The practical seminar was conducted in medium-sized groups (about 20. students) on patients (divided into 4 patients) and seminar rooms. The block practical was conducted in small groups (8 students) directly on the patient.

To allow all students to participate in the digital teaching, all students were granted access to the required software via the student email address, which is assigned at the beginning of their study.

Due to the Covid-19 pandemic, the main lecture was converted to a purely digital lecture. The lecture was pre-recorded by the teacher in advance. The usual lecture slides were used and explained by the lecturer’s comments. The corresponding lecture could be accessed by the students via the teaching platform Moodle, paused and repeated as often as desired. The lectures could thus be used largely unchanged.

The practical seminar and block internship were held as webinars instead of face-to-face small group classes. The Microsoft Teams TM communication software was used for this purpose. The character of the small group instruction was preserved, and the number of participants was not changed. The subject matter was presented in an interactive lecture. The possibility to ask questions and to promote a discussion in the form of problem-oriented learning (POL) was actively encouraged. The physical examination techniques were described in detail by the lecturers with special attention to all movement steps and possible sources of error. The small group teaching was designed bidirectionally. Regular question rounds and clarification of the examination techniques on clinical examples ensured individual knowledge transfer. At the end of the webinar there was always an open question round, which gave the opportunity to clarify open and unclear issues.

The statistical analyses were performed with GraphPad Prism 9.

## Results

### Baseline characteristics

258 students participated in the corresponding student courses. A total of 192 students completed the questionnaire (75.89% response rate). Among the total 192 included students, 109 (56.8%) were in the main lecture and practical seminar (7th semester) and 83 (43.23%) were in the block practical (8th semester). Among them, a total of 133 were female (69.27%), 57 were male 29.69%) and 2 were nonbinary (1.04%). The majority of students were between 18 and 25 years old (138 students, 71.88%). 38 students were between 26 and 30 years old (19.8%) and 16 were over 30 years old (8.3%) (Table 1).

**Table 1:** Baseline characteristics. Data in percent of responses

**Attended course (192 responses)**	
Main lecture/practical seminar (7th semester)	56.77%
Block internship (8th semester)	43.23%
**Gender of the participants (192 answers)**	
Female	69.27%
Male	29.69%
Nonbinary	1.04%
**Age of the participants (192 answers)**	
18–25 years	71.88%
26–30 years	19.79%
> 30 years	8.33%

### Surgical skills and surgical career

**Fig. 1:**
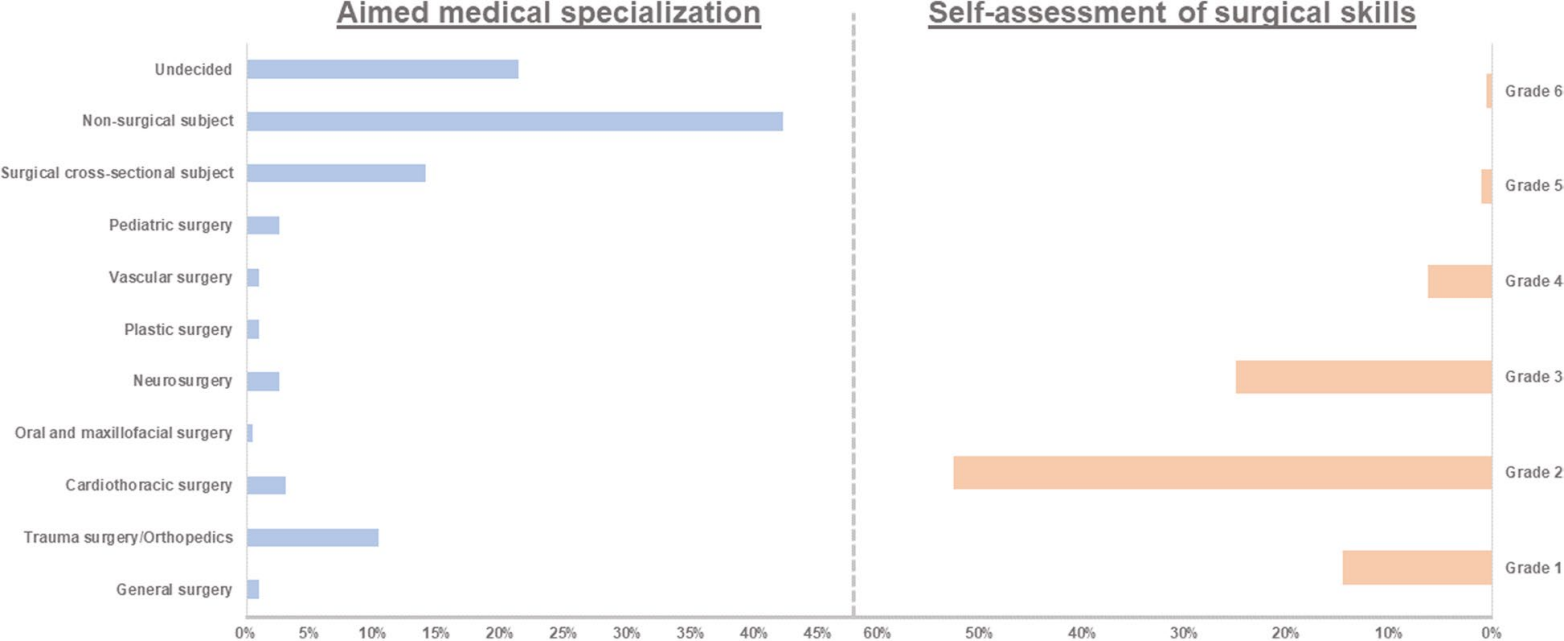
The left-hand side (blue bars) shows the desired medical training that the students are aiming for. Indication in percent. The right-hand side (red bars) shows the students’ own assessment of their surgical skills. Indication in percent. From grade 1 (best) to grade 6 (worst),. Left: self-assessment of manual/surgical skills. Indication in grades. Grade 1 (best) to 6 (worst). Data in percent of 192 students. Right: Aimed medical specialization. Data given as a percentage of 192 students. Surgical cross-section includes specialties such as urology and gynecology

Among the students, 36.46% wanted to pursue an operative specialty as a career goal. Of those, 27 (14.1%) wanted to target a cross-sectional surgical specialty (e.g. urology and gynecology). 20 (10.4%) were interested in trauma surgery and orthopedics. 5 (2.6%) each for the subject’s pediatric surgery and neurosurgery. 4 (2.1%) students wanted to practice cardiothoracic surgery. The majority of students (81 students, 42.2%) plan to continue their residency training outside of surgery in the future. 41 students (21.4%) are still undecided about their future profession (Fig. 1).

On average (52.6%), students rate their manual ability as good (grade 2). 14.58% even rate this as very good (grade 2). 25% feel their manual ability is satisfactory (grade 3). 7.81% think their craft skills are sufficient to insufficient (grade 4–6) (Fig. 1). Here it can be observed that the intended specialty had no relevant effect on the self-assessment (*p* = 0.2). Thus, surgically interested students did not rate their manual skills better than the non-surgically interested students.

### Structure and expectation of the eLearning

Through the eLearning events, 94 students had wished to learn more about the subject of surgery and 85 students had hoped to develop their practical skills. 12 students wanted the courses to facilitate their future career choice. 1 student did not answer the question.

Students rated clinical relevance an average of 4.26 out of a possible 6 points (4 students gave 1 out of 6 points, 10 students gave 2 out of 6 points, 31 students gave 3 out of 6 points, 59 students gave 4 out of 6 points, 62 students gave 5 out of 6 points, 25 students gave 6 out of 6 points). 1 student did not answer the question.

The vast majority of students felt that the student requirement of the eLearning events was well-designed (144 students, 75.79%).

Students rated the structural design with an average of 3.49 out of a possible 6 points (25 students gave 1 out of 6 points, 22 students gave 2 out of 6 points, 37 students gave 3 out of 6 points, 56 students gave 4 out of 6 points, 36 students gave 5 out of 6 points, 12 students gave 6 out of 6 points). However, over half of all students feel that the structural design is too teacher dependent (55.32%).

### Used software

The course management system used, Moodle, was rated an average of 4.61 out of a possible 6 points (6 students gave 1 out of 6 points, 11 students gave 2 out of 6 points, 18 students gave 3 out of 6 points, 39 students gave 4 out of 6 points, 61 students gave 5 out of 6 points, 57 students gave 6 out of 6 points).

The chat and video conferencing program used, Microsoft Teams, was given an average rating of 4.92 out of a possible 6 points (5 students gave 1 out of 6 points, 4 students gave 2 out of 6 points, 17 students gave 3 out of 6 points, 26 students gave 4 out of 6 points, 36 students gave 5 out of 6 points, 77 students gave 6 out of 6 points).

The Digital Textbook and Exam Preparation Program Amboss (https://www.amboss.com) was considered useful and sufficient by 161 of 191 students (one abstention).

### eLearning in the future

73.68% of the participating students consider that eLearning lectures should continue to be offered. 26.32% of the participating students are of the opinion that teaching should continue as usual (face-to-face lectures).

Whether an eLearning lecture is more useful than a face-to-face event, however, is the opinion of only 54.17% of the students. 45.83%, on contrast, find a face-to-face event to be more beneficial.

Even though the majority of students perceives eLearning a useful addition, a plurality of students (59.38%, 114 students) is against a purely virtual university (attendance with the help of an avatar). 40.63% of the surveyed are open to the idea of a virtual university.

Among students pursuing operational careers, 40.85% were open to a digital-only university. Among students who preferred a non-operational subject, the figure was 46.34%.

**Fig. 2:**
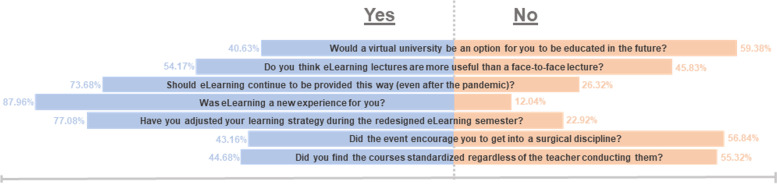
Graphical representation of the students’ answers. The question is shown in the middle of the bars. The left side (blue bars) shows how many students answered yes to the question. The right side (red bars) shows how many students answered no to the question. To the left and right of the bars is the exact percentage. Illustration of some answers. The corresponding question is shown in the middle of the bars. Left (blue bar) shows in percent how often the question was answered with yes. On the right (orange bar) shows in percent how often the question was answered with no. The exact percentage is shown next to the bars

Figure 2 illustrates the responses of some of the questions.

### Quality of teaching

The teaching quality was rated on average 3.69 out of 6 possible points (19 students gave 1 out of 6 points, 19 students gave 2 out of 6 points, 36 students gave 3 out of 6 points, 57 students gave 4 out of 6 points, 50 students gave 5 out of 6 points, 11 students gave 6 out of 6 points).

## Discussion

The Covid-19 outbreak and resulting pandemic has brought much of social life to a shutdown. In particular, systemically important segments such as medical care require a steady supply of well-trained professionals, which cannot be sustained without a structured continuation of education.

Classroom lectures are crucial for continuing education in a subject with a strong practical orientation such as surgery. These could not be held in order to protect the health of the students and the society. In attempt to continue structured teaching, new ways had to be found to ensure this. In the context of the viral pandemic, digitized learning content and lectures for surgical training of medical students seem promising.

Nowadays, varieties of technical opportunities for eLearning are available. Webinars are effective tools in medical teaching [12–14] and have the advantage of continuing to provide problem-based learning in small groups. Bidirectional communication, which is essential for learning surgical knowledge and skills, can be maintained to a certain extent. The lectures, which primarily serve to provide theoretical knowledge, have been replaced by pre-recorded on demand lectures. Even if the original indirect interaction between lecturers and students is missing [15] the recorded lectures have some advantages. It is up to the students when and how often the lectures are processed. In addition, pausing the lectures allows students to adapt to their own learning speed [16].

Alternatively, theoretical and practical knowledge could be transferred into a pure digital world using virtual reality (VR) to simulate a practical teaching environment [17, 18]. Also of interest is the usage of Augmented reality (AR) which adds virtual aspects and information to the physical environment [19]. Even though the technical aspect of VR and AR has become much more affordable in recent years [20], an immediate implementation is difficult for a large number of students and risks excluding some students. To provide an as fast as possible implementable, cost-efficient and area-wide teaching concept we have switched surgical teaching to already partially established webinars and remote lectures.

To our surprise, the switch to eLearning was the first contact with digital learning for a large number of students. Nevertheless, it led to a large acceptance of new media. Even beyond the COVID-19 pandemic, the majority of students would like to see a continuation of digital education offerings. These findings are consistent with other studies in the medical sector [21–23]. This could be explained by the improved flexibility and the elimination of otherwise necessary commuting distances [24, 25]. However, almost half of the students consider face-to-face courses to be more beneficial. It should be noted that the acceptance and success of eLearning is still not solely dependent on the teaching method used [26, 27]. Hence, the desire for face-to-face courses may continue to decline in the course of improved teaching skills and adaptation of the curriculum to digital education. Thus, 40% of students are now already open to the idea of a purely digital university. Wherein, regardless of further didactic development, hybrid teaching concepts consisting of face-to-face courses and digital teaching tools such as webinars, AR/VR and remote lectures can have a synergistic effect on teaching in general medical practice. Here, in addition to the digitally imparted theoretical knowledge, hardskills such as examination techniques, operation assistance, and basic surgical manual skills could be attained.

Even if the theoretical knowledge could be learned through the digital restructuring, the learning of practical skills is difficult to implement. This is especially unfortunate given the students’ desire to improve their surgical skills. In the event that the prevailing condition of contact restrictions is sustained beyond a longer period of time, new formats for teaching the above-mentioned practical skills are urgently needed, irrespective of the approaches that have already been developed [28, 29].

Overall, the requirements for eLearning were well designed, so that the necessary teaching content could also be conveyed via eLearning. Here, the clinical relevance was indicated by the studs with an above-average success. These results are in line with studies that had a similar concept in face-to-face courses [30]. In addition, the structural design was rated as beneficial by the students. Previous studies have already shown that e-learning is on a level with face-to-face teaching in terms of quality. In these studies, digitization resulted in equal or even better grades in the final exams [31, 32]. Such fixed endpoints were not examined in the present study. Whether the digital courses can also impart surgical knowledge in the long term remains to be clarified by further studies.

The study is subject to certain limitations. A comparative study should be contrasted with a control group if possible. However, due to the pandemic, no students were able to attend conventional face-to-face classes at the time of the survey, and therefore no comparison was possible. In the future, a study is planned which will allow an appropriate comparison. The end-user experience often depends on the end-user platform used (tablet, computer smartphone) and can therefore lead to a distortion of the findings.

## Conclusion

In this study, we examined the student experience of converting conventional surgical teaching to eLearning. For a large proportion of students, the conversion represented their first contact with eLearning and was accepted predominantly positively. In order to continue surgical teaching digitally over a long period of time, new digital formats for teaching hard skills need to be developed and implemented.

## Supplementary Information

**Additional file 1: Attachment 1.** Overview of the survey. Under the boldly written questions the answers are given.

## Data Availability

The datasets used and analysed during the current study are available from the corresponding author on reasonable request.

## Abbreviations

VR: Virtual reality
AR: Augmented reality
