# Supplementary material for: COVID-19: a catalyst for the digitization of surgical teaching at a German University Hospital
Source: BMC Med Educ. 2022 Apr 22;22:308. doi: 10.1186/s12909-022-03362-2 (PMC9030685; doi:10.1186/s12909-022-03362-2)
Supplement: Supplementary file 1 — Additional file 1: Attachment 1. Overview of the survey. Under the boldly written questions the answers are given. [file 12909_2022_3362_MOESM1_ESM.docx]

Attachment 1: Overview of the survey. Under the boldly written questions the answers are given.

| **Which course did you attend?** | | | | | |
| --- | --- | --- | --- | --- | --- |
| Block practical | | | Main lecture and practical seminar | | |
| **Please enter your gender.** | | | | | |
| Female | | Male | | Divers | |
| **In which age category do you assign yourself? (Age in years)** | | | | | |
| 18-25 | | 26-30 | | >30 | |
| **How would you rate your manual skills? (Indicated in school grades; 1= best, 6=worst)** | | | | | |
| 1 | 2 | 3 | 4 | 5 | 6 |
| **Please indicate your professional goal.** | | | | | |
| General surgery | | Trauma surgery/Orthopedics | | Cardiothoracic surgery | |
| Oral and maxillofacial surgery | | Neurosurgery | | Plastic surgery | |
| Pediatric surgery | | Vascular surgery | | Undecided | |
| Non-surgical subject | | | Surgical cross-sectional subject (e.g., urology or gynecology) | | |
| **What were your expectations for the event?** | | | | | |
| Learn more about the subject surgery. | | To simplify my later career choice. | | Learn practical surgical skills. | |
| **How did you experience the requirements of the event?** | | | | | |
| Overloaded | | Just right | | Underwhelmed | |
| **Did you consider the event to be structured? (Indicated in points, 1=very bad, 6=very good)** | | | | | |
| 1 | 2 | 3 | 4 | 5 | 6 |
| **How would they rate the clinical relevance of the course? (Indicated in points, 1=very bad, 6=very good)** | | | | | |
| 1 | 2 | 3 | 4 | 5 | 6 |
| **Did you find the event standardized regardless of the teacher presenting it?** | | | | | |
| Yes | | | No | | |
| **Did the event encourage you to get excited about a surgical specialty?** | | | | | |
| Yes | | | No | | |
| **Have you adjusted your learning strategy during the redesigned eLearning semester?** | | | | | |
| Yes | | | No | | |
| **Was eLearning a new experience for you?** | | | | | |
| Yes | | | No | | |
| **How did you rate the online platform "Moodle"? (Indicated in points, 1=very bad, 6=very good)** | | | | | |
| 1 | 2 | 3 | 4 | 5 | 6 |
| **How did you rate the use of MS teams for the webinars? (Indicated in points, 1=very bad, 6=very good)** | | | | | |
| 1 | 2 | 3 | 4 | 5 | 6 |
| **Was the selected AMBOSS content sufficient for you?** | | | | | |
| Yes | | | No | | |
| **Do you think the eLearning lectures are more useful than a face-to-face lecture?** | | | | | |
| Yes | | | No | | |
| **Would a virtual university be an option for you to be educated in the future? (Attendance with the help of an avatar)?** | | | | | |
| Yes | | | No | | |
| **Should eLearning continue to be provided this way (even after the pandemic)?** | | | | | |
| Yes | | | No | | |
| **How would you rate the quality of teaching this semester? (Indicated in points, 1=very bad, 6=very good)** | | | | | |
| 1 | 2 | 3 | 4 | 5 | 6 |
